# Biodegradable Magnesium‐Based Implants in Orthopedics—A General Review and Perspectives

**DOI:** 10.1002/advs.201902443

**Published:** 2020-02-28

**Authors:** Jia‐Li Wang, Jian‐Kun Xu, Chelsea Hopkins, Dick Ho‐Kiu Chow, Ling Qin

**Affiliations:** ^1^ School of Biomedical Engineering Sun Yat‐sen University Guangzhou 510006 P. R. China; ^2^ Musculoskeletal Research Laboratory Department of Orthopaedics & Traumatology The Chinese University of Hong Kong Hong Kong SAR P. R. China; ^3^ Innovative Orthopaedic Biomaterial and Drug Translational Research Laboratory Li Ka Shing Institute of Health Sciences The Chinese University of Hong Kong Hong Kong SAR P. R. China

**Keywords:** challenging bone diseases, magnesium, orthopedic implants, surface modification, weight‐bearing

## Abstract

Biodegradable Mg‐based metals may be promising orthopedic implants for treating challenging bone diseases, attributed to their desirable mechanical and osteopromotive properties. This Review summarizes the current status and future research trends for Mg‐based orthopedic implants. First, the properties between Mg‐based implants and traditional orthopedic implants are compared on the following aspects: in vitro and in vivo degradation mechanisms of Mg‐based implants, peri‐implant bone responses, the fate of the degradation products, and the cellular and molecular mechanisms underlying the beneficial effects of Mg ions on osteogenesis. Then, the preclinical studies conducted at the low weight bearing sites of animals are introduced. The innovative strategies (for example, via designing Mg‐containing hybrid systems) are discussed to address the limitations of Mg‐based metals prior to their clinical applications at weight‐bearing sites. Finally, the available clinical studies are summarized and the challenges and perspectives of Mg‐based orthopedic implants are discussed. Taken together, the progress made on the development of Mg‐based implants in basic, translational, and clinical research has laid down a foundation for developing a new era in the treatment of challenging and prevalent bone diseases.

## Graphical abstract

This Review summarizes the current status and future research trends for Mg‐based or containing orthopedic implants. The progress made on the development of Mg‐based or containing implants in basic, translational, and clinical research can lay down a foundation for opening a new era to improve clinical healing outcomes in the treatment of challenging bone diseases.

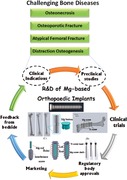

## 1 Introduction

There is an increasing demand on innovative clinical orthopedic implants for ageing‐related bone diseases including but not limited to osteoporotic fracture.^1^ Traditional orthopedic implants including fixators (internal/external) and prosthesis are made of inert stainless steel, titanium (Ti) or its alloys, and cobalt–chromium (Co–Cr) alloys. They possess satisfactory biocompatibility, high wear resistance, and adequate mechanical strength.^2^ However, there are known limitations for such implants when applied in fracture fixation. First, the long‐term implantation of bioinert metallic devices may permanently capture the drilled hole and thus may produce extra difficulties in case of revision surgery is required. Second, the stress shielding effects elicited by the high Young's modulus of current metals may cause peri‐implant bone loss over time, ultimately leading to deterioration of fixation that often ends up with revision surgery.^3^ Third, if there are unexpected clinical complications, such as pain or impaired function, a second surgery for implant removal is required.^4^ In addition, these traditional metals severely affect the diagnostic accuracy of X‐ray and computed tomography (CT) images, due to the beam hardening and its associated imaging artefacts.^5^

Synthetic polymers are important alternative materials to traditional metallic counterparts in orthopedics at low weight‐bearing skeletal sites.^6^, ^7^ Ultrahigh Molecular Weight Polyethylene (UHMWPE), poly(methylmethacrylate) (PMMA), polyurethanes (PU), and polyetheretherketone (PEEK) are the most commonly used nondegradable polymers approved by United States Food and Drug Administration (US FDA) for orthopedic application,^8^ while poly(l‐ or d,l‐lactic acid), poly(glycolic acid), and polycaprolactones (PCL) are widely used absorbable polymers as orthopedic fixation implants with US FDA approval.^6^ The nondegradable polymers are preferable to metal devices, as they do not cause artefacts during radiographic imaging and there is a low re‐fracture risk due to the absence of stress shielding. However, the toxic residual monomers and wear debris from these nondegradable polymer implants may easily induce undesirable effects, eliciting increasing clinical concerns.^9^ The resorbable polymers have similar advantages as those in nondegradable polymers. More importantly, the monomers released from the resorbable polymers during in vivo degradation show high biocompatibility, so they have been extensively applied in orthopedics.^9^ However, bulk erosion of resorbable polymers leads to accumulation of the acidic intermediate degradation products, which may induce a noninfectious inflammatory reaction, contributing to pathological bone resorption.^10^ Natural polymers, such as collagen and chitosan, demonstrate better biocompatibility, but there are no US FDA approved orthopedic fixators because of their poor mechanical properties, possible antigens that could lead to inflammatory reactions, and the difficulties in processing.^11^

Although the traditional metallic and synthetic polymeric orthopedic implants have been predominantly applied in surgeries, they have shown increasing limitations in the treatment of some challenging bone diseases including osteoporotic fractures,^12^ nontraumatic osteonecrosis,^13^ atypical femoral fractures,^14^ and distraction osteogenesis^15^ due to impaired osteogenesis and angiogenesis of the host bone tissue.^14^, ^16^ Recently, there have been enormous studies about the development of magnesium (Mg)‐based orthopedic implants dedicated by material engineers, preclinical scientists and clinicians,^17^, ^18^, ^19^, ^20^ which may be able to address the flaws in current commercialized orthopedic implants. Mg is a biodegradable metal, with good biocompatibility and desirable Young's modulus close to that of natural cortical bone, and thus widely recognized as a potentially revolutionary orthopedic biomaterial.^21^ More importantly, increasing evidences demonstrate that Mg ions released from Mg‐based implants after surgical implantation in vivo can promote bone regeneration and accelerate healing in bone diseases.^22^, ^23^ The Mg‐based orthopedic implants, which exert beneficial effects on the formation of new blood vessels and bone tissue,^18^, ^22^ may provide a distinct advantage over non‐Mg‐based counterparts for treatment of the challenging bone disorders.

Although there are numerus Reviews focusing on the design and degradation pattern of biodegradable metals,^24^, ^25^ testing methods of corrosion mode of Mg‐based metals,^21^ current reported clinical trials,^20^ and interaction between biomaterials and cells,^26^ it still remains unclear with regards to the repair mechanism of Mg ions in bone fracture repair, extension of future clinical indications, and strategies for its application at high weight‐bearing skeletal sites. Therefore, we would like to address these questions in this Review and hope to broaden our understanding on its basic medical sciences and extend the use of Mg‐based implants in orthopedics.

## 2 Advantages of Mg‐Based Metals for Orthopedic Applications

### 2.1 Unique Mechanical Properties of Mg

**Figure 1:**
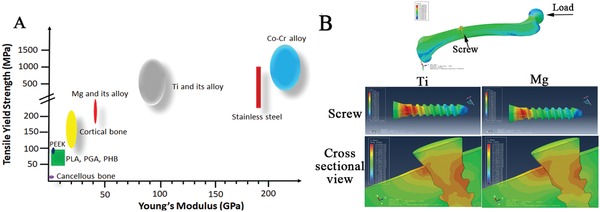
Mg and its alloys possess Young's modulus close to natural bone as compared to the traditional metallic orthopedic devices. A) The distribution range of tensile yield strength of natural bone, commercially available orthopedic implants made of polymers and inert metals, and biodegradable Mg and its alloys; B) The peak stress distribution in a finite element analysis (FEA) model composed of a femur and an inserted titanium (Ti) or Mg‐based screw. Higher stress distribution is observed within the Ti screw as compared to the Mg‐based screw, relative to the lower stress distribution in the peri‐screw bone tissue, indicating stress shielding at the implant‐bone interface in the Ti group. PEEK: polyetheretherketone; PLA: poly(lactic acid); PGA: poly(glycolic acid); PHB: polyhydroxybutyrate; Co–Cr: Cobalt–Chromium.

Mg and its alloys have a higher strength relative to natural bone, but the Young's modulus closely matches that of cortical bone, implying it unique feature on reducing stress shielding during load transfer at the interface of implant to bone (**Figure** **1**). These properties therefore overcome the shortcomings of traditional metallic and synthetic polymeric orthopedic devices, making it a more suitable candidate for treating the challenging bone diseases.^17^

### 2.2 High Biocompatibility of Mg during Degradation in Human

**Figure 2:**
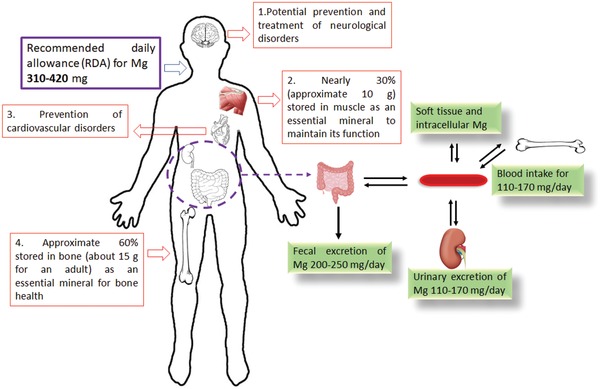
Good biocompatibility of biodegradable Mg‐based orthopedic implants. The Mg ions released from Mg‐based orthopedic implants can be promptly and effectively diluted by the body fluid and transported to other soft tissues and bones via blood or tissue fluid circulation. Excessive Mg ions are excreted via urine and feces.^29^, ^30^

The definition of an absorbable metallic material by American Society for Testing and Materials (ASTM)‐F3160 in 2016, states: “an initially distinct foreign material or substance that either directly or through intended degradation can pass through or be metabolized or assimilated by cells and/or tissue.”^27^ Mg metal has a lower standard electrode potential than hydrogen and can be degraded into Mg ions and hydrogen gas in an aqueous solution under standard conditions.^28^ As the fourth most prevalent mineral in the human body, Mg is involved in hundreds of biochemical reactions and acts as an essential element in the construction of bone and soft tissue.^29^ A healthy adult restores 24–30 g of Mg for maintaining regular functions and the recommended daily allowance (RDA) for Mg is 310–420 mg to maintain health.^29^ Excessive Mg ions are permissible as they can be transported via the circulatory system and promptly excreted by way of urine and faces, without causing any adverse effects^30^ (**Figure** **2**). Thus, Mg‐based metals can be defined as a type of novel absorbable metallic material.

**Figure 3:**
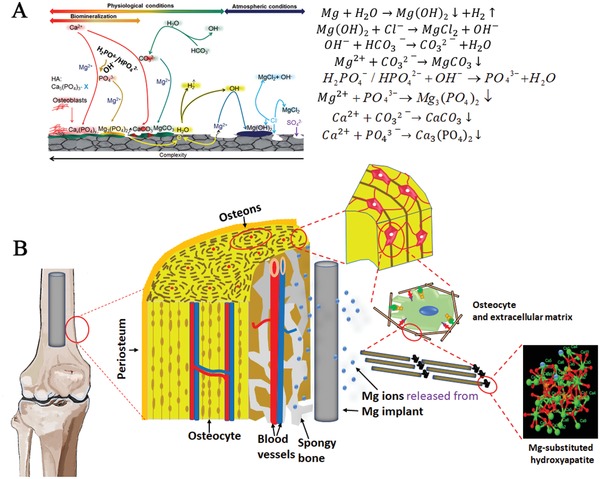
Degradation mechanisms and diffusion patterns of Mg‐based orthopaedic implants. A) Degradation behaviour of Mg‐based orthopaedic implants and the corrosion products of Mg‐based orthopaedic devices under physiological conditions. Adapted with permission.^32^ Copyright 2018, Elsevier. B) Proposed diffusion of released Mg ions from the Mg‐based orthopaedic devices into the extracellular matrix to form Mg‐substituted hydroxyapatite in bone tissue.^34^

The presence of inorganic salts in the aqueous solutions of the human body complicates the degradation of Mg‐based metals (**Figure** **3**A). The main intermediate product deposits on the surface of Mg‐based metals are: Mg(OH)_2_, MgCO_3_, Mg_3_(PO_4_)_2_, CaCO_3_, and Ca_3_(PO_4_)_2._ 31, 32 In situ observation demonstrates that these deposits can be phagocytosed by macrophages.^33^ Furthermore, synchrotron microbeam X‐ray fluorescence (μXRF) mapping demonstrates that the Mg ions released from the degradation of the intermediate products are temporarily stored in the bone matrix,^34^ partially contributing to the fate of the released Mg ions. Osteoporotic patients usually suffer from Mg deficiency,^35^ leading to larger crystal size and higher crystallinity in HAP, so the osteoporotic bone becomes fragile and brittle.^36^ Small angle X‐ray scattering (μSAXS) and X‐ray diffraction (μXRD) measurements show that the inclusion of Mg in HAP reduces the crystal size and the crystalline order due to the replacement of Ca by Mg^34^ (Figure 3B), so the use of the Mg‐based implants may improve the new bone strength at the peri‐implant site.^34^ As the extracellular inorganic matrix, the Mg‐substituted HAP shows highly bioactivity and enhanced osteoconductivity and osseointegration.^37^ With the degradation of Mg‐based implants, the temporarily stored Mg ions in the peri‐implant bone matrix can be then gradually released into circulation system without affecting blood serum level of Mg ion concentration.^34^ As a novel antioxidant, the hydrogen (H_2_) gas, which is accompanied with the production of Mg ions, could attenuate oxidative stress‐induced senescence process during mesenchymal stem cell (MSC) expansion,^38^ contributing to prevention of bone loss in osteoporosis group.^39^ Although H_2_ gas can be exchanged quickly in the local tissue via measuring H_2_ concentration,^40^ it is still possible to form the gas bubble in the peri‐implant site if the degradation rate of the Mg‐based implants becomes faster, which might cause health risks in patients according to the reported studies performed in rats.^41^ Therefore, the control of the degradation rate of the Mg‐based implants has been considered as an effective strategy to reduce H_2_ accumulation.^42^, ^43^ Besides, hydroxide ions (OH^−^) is also accompanied, but the alkaline condition (higher pH value) adjacent to the Mg‐based implants in vivo could be quickly neutralized according to the in situ measurement using a micro‐pH sensor.^44^

### 2.3 Osteopromotive Properties of Magnesium

**Figure 4:**
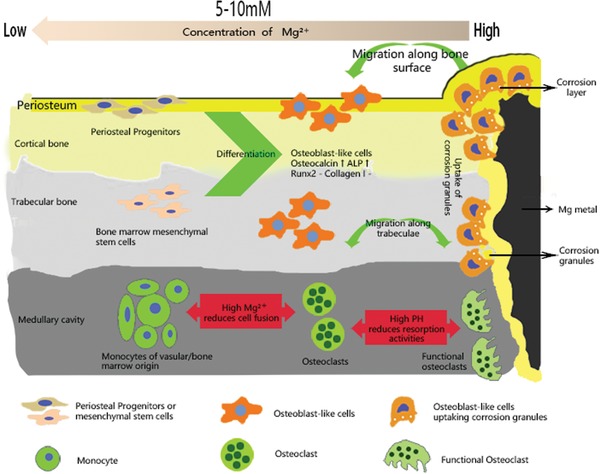
Schematic diagram showing the tissue and cellular responses to the Mg and pH gradients after implantation of Mg‐based screws. Both bone marrow stem cells (BMSCs) and periosteal stem cells (PSCs) differentiate into osteoblast‐like cells and migrate to the implantation site to remove the corrosion granules. In contrast, both high Mg ion concentration and pH reduces the fusion of pre‐osteoclasts, thereby inhibiting osteoclastogenesis. This biological mechanism underlines the beneficial effects observed in anterior cruciate ligament (ACL) reconstruction and plate‐screw fixed bone‐fracture at metaphyseal region.^46^, ^51^, ^52^ Reproduced under the terms of the Creative Commons CC BY license.^50^ Copyright 2018, Springer Nature.

**Figure 5:**
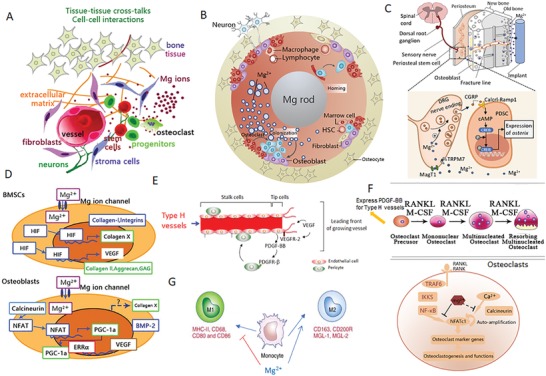
Cellular and molecular mechanisms demonstrating the potential benefits of Mg ions on bone homeostasis. A) Schematic diagram showing the stimulation of Mg ions (released from Mg‐based implants) on the cross‐talk between connecting tissues (bone, nerves, and vessels), as well as the interactions between cells (stem cells, osteoblast, osteocytes, osteoclasts, endothelial cells, and macrophages); B) Cross‐sectional view of the cellular components affected by the release of Mg ions from an intramedullary orthopaedic implant; C) Robust bone formation at the periosteal region demonstrating the differentiation of periosteum stem cells (PSCs) through the activation of Mg‐induced calcitonin gene‐related peptide (CGRP). This further demonstrates the underlying mechanism that improves healing of osteoporotic bone fractures at the femoral mid‐shaft. Reproduced with permission.^18^ Copyright 2016, Nature. D) Mg ions directly promote the expression of hypoxia‐induced factor (HIF) in bone marrow mesenchymal stem cells (BMSCs), leading to enhanced chondrogenesis (increased collagen II, aggrecan, and collagen X) and osteogenesis (increased collagen I, BMP‐2, and integrins). Adapted with permission.^53^ Copyright 2014, Elsevier. E) Mg‐induced production of vascular endothelial growth factor (VEGF) is an essential factor for neo‐formation of type H (CD31^hi^Endomucin^hi^) vessels, which may regulate bone homeostasis. Reproduced with permission.^129^ Copyright 2014, Atlas of Genetics and Cytogenetics in Oncology and Haematology. F) Mg ions inhibit the differentiation of osteoclast precursors by suppressing NF‐κB and NFATc1. High endogenous expression of PDGF‐BB in osteoclast precursors may enhance type H vessel formation. Adapted with permission.^52^ Copyright 2014, Elsevier. G) Mg ions may promote macrophages to polarize toward the M2 phase that promotes tissue regeneration, instead of M1 phase that promotes an inflammatory response.

Zheng et al.^24^ previously defined Mg‐based metals as a type of “biodegradable metal” from the aspect of biofunction of materials owing to the osteopromotive effects of their degradation products.^18^, ^45^, ^46^ Bone and its surrounding microenvironment constitute a sophisticated system consisting of multiple stem cells, progenitors, osteocytes, osteoblasts, osteoclasts, neuronal fibers, endothelial cells (in circulatory vessels), and immune cells. To date, the effects of Mg on these cells have been comprehensively investigated using both in vitro and in vivo models (**Figures** **4** and **5**). The responsible cell types (components) largely depend on the region receiving implantation (that is, metaphysis or diaphysis). For example, periosteum is important for the repair of diaphysis while less involved in the metaphyseal region where BMSCs contributed more to the formation of trabecular bone.

When Mg‐based screws were used to fix the tendon graft in the drilled bone tunnel after anterior cruciate ligament (ACL) reconstruction, the surrounding trabecular bone quality was greatly improved,^47^, ^48^, ^49^ most likely attributed to the high concentration of Mg ions (>15 × 10^−3^ m) and high pH, which reduces the fusion of pre‐osteoclast cells and the activity of osteoclasts (Figure 4), thereby inhibiting osteoclastogenesis.^50^ Consistently, Mg screw also improved healing in a femoral intra‐condylar fracture model, demonstrating with better osseointegration and increased bone volume and bone mineral density at fracture gap, as compared to those fixed with PLA screws.^51^

When Mg‐based nails are implanted into the marrow cavity of long bones, e.g., femur, Mg ions can be released with implantation over time. There are two pathways for the diffuse of the released Mg ions. The first one is along the natural (Harversian and Volkmann's) or artificial (the bone‐fracture gaps/lines) canals. The other one is from the marrow to the periosteal region that is densely occupied by periosteum stem cells (PSCs) and sensory neuron fibers (Figure 5A–C).^18^ Through these avenues, our innovative Mg‐containing intramedullary nail significantly improved femoral fracture in an established osteoporotic animal model in rats, shedding light on the Mg‐orchestrated connection between the sensory nerve system and PSCs.^18^, ^56^ Chaya and colleagues^57^ also confirmed the feasibility on the application of Mg plate and screws in a rabbit ulna fracture model as presented with comparable bending strength to that of intact ulna, at week 16 post healing. On top of these findings, we further developed Mg/Ti hybrid fixation system to fix a “Z‐shaped” open osteotomy at the mid‐shaft of rabbit tibia.^45^ Compared to the pure Ti fixation, faster endochondral ossification with higher osteocalcin improved mechanical strength when combining with Mg screws.^45^ Here we discuss the cellular and molecular mechanisms behind the beneficial effects of Mg in details.

#### 2.3.1 Cross‐Talk between Sensory Nerves and PSCs

Inspired by previous literatures reporting that Mg ions promoted the CGRP secretion in women with preeclampsia,^58^ we further found that transport of Mg ions into the neurons in the dorsal root ganglions was mediated by Mg transporter 1 (MagT1) and Transient Receptor Potential cation channel subfamily member 7 (TRPM7) which promoted the release of Calcitonin Gene‐Related Peptide (CGRP).^18^, ^56^ As shown in Figure 5C, CGRP further binds to its receptor expressed on the surface of PSCs, triggering the binding of cyclic adenosine monophosphate (cAMP) to its response element binding protein (CREB). This leads to elevation of osterix (SP7), which robustly stimulates new bone formation, especially at the periosteal region. His at least, helps us explain the healing improvements in femoral fracture of osteoporotic rats^18^ and tibial fracture in rabbits.^45^

#### 2.3.2 Mg‐Promoted Osteogenic Differentiation

Yoshizawa et al. reported that an addition of 10 × 10^−3^ m Mg ions to cell cultures of human bone marrow mesenchymal stem cells (hBMSCs) and differentiating osteoblasts (Figure 5D), enhanced the mineralization of the extracellular matrix (ECM) by increasing the production of collagen‐X and vascular endothelial growth factor (VEGF).^53^ They further demonstrated that Mg‐elevated VEGF was jointly regulated by hypoxia inducible factor 2a (HIF‐2a) and peroxisome proliferator‐activated receptor gamma coactivator (PGC)‐1a.^53^ In addition, in BMSCs, Mg ions at this dose significantly activated integrins (α5β1)^59^ and focal adhesion kinase pathways,^47^ which are important for osteogenesis.^60^ Recently, Hung et al. found that the protein expression of active β‐catenin was increased in hBMSCs when treated with 10  × 10^−3^ m  Mg ions, accompanied with the increased expression of LEF1 and Dkk1, suggesting the activation of canonical Wnt signaling pathway.^61^ Altogether, these molecular events help understand the contributions from BMSCs and their descendants, which are crucial in particular for the trabecular regions.

#### 2.3.3 Mg‐Enhanced Angiogenesis May Couple Bone Formation

Given VEGF was enhanced by Mg as mentioned above, and VEGF plays a central role in the development of vessels including type H capillaries (Figure 5E) which are essential for bone formation,^55^, ^62^ we could not exclude the potential contributions from Mg‐enhanced angiogenesis, by contrast further supporting by the fact that Mg is able to directly promote vessel growth under ischemic condition.^63^ Indeed, either bone cements or PLGA scaffold containing Mg improved both osteogenesis and angiogenesis,^64^, ^65^ even in the challenging osteonecrotic bone defect model.^65^ Furthermore, Mg‐enriched microspheres not only stimulated the osteogenic differentiation of stem cells but also promoted neovascularization.^66^ However, the cell sources and molecules on this aspect require further investigation.

#### 2.3.4 Mg‐Inhibited Osteoclastogenesis May Enhance Angiogenesis and Osteogenesis

Mg^2+^ deficiency augments osteoclastogenesis.^67^ In contrast, Zhai et al. found that supplementation of Mg ions could prevent wear particle‐induced osteolysis in vivo via inhibition of nuclear factor of activated T‐cells cytoplasmic 1 (NFATc1) and activation of nuclear factor‐kB (NF‐κB) (Figure 5F), suggesting that Mg has an anti‐osteoclastogenic effect.^52^ This provides a source of osteoclastic progenitors that secrete platelet‐derived growth factor‐BB (PDGF‐BB), which may support type H capillary growth.^54^ Together with the proven elevation of PDGF‐BB in peri‐implant tissue during the degradation of Mg implants,^47^ it is reasonable to propose the cross‐talk between osteoclastogenesis and angiogenesis.

On the other hand, to better understand the in vivo interactions, a coculture system consisting of human osteoblasts and osteoclasts was established.^68^ In vitro experiments using this coculture model further support the finding that Mg promotes osteogenesis while inhibits osteoclastogenesis.^68^

#### 2.3.5 Osteoimmunomodulatory Effect

Osteoimmunological responses are particularly emphasized for the bone formation and fracture healing.^69^, ^70^ It has recently been established that Mg‐based biomaterials can induce an innate immune response in macrophages (Figure 5G).^64^, ^71^ Mg promotes macrophage polarisation to the M2 phase, which supports osteoblast mineralization,^71^, ^72^ and suppresses the M1 phase, which supports an inflammatory response, thus demonstrating that Mg may also act as an anti‐inflammatory agent.^73^ This anti‐inflammatory effect is likely at least attributed to Mg‐regulated TRMP7.^74^ Followed by promoting macrophages polarize toward M2 phase, Mg‐coated Ti also up‐regulated BMP2 and VEGF,^72^ while down‐regulated the NF‐κB signaling.^72^

Therefore, Mg plays multifunctions in bone growth and regeneration. It exerts both direct and indirect effects between connecting bone, vessel, nerve, and immune systems, creating potential for functional bone regeneration. These cellular and molecular mechanisms together contribute to the enhanced healing of long bone fractures in animal models, including osteoporotic animal models.^18^, ^57^, ^75^

## 3 Animal Models for Studying Mg‐Based Orthopedic Implants

**Figure 6:**
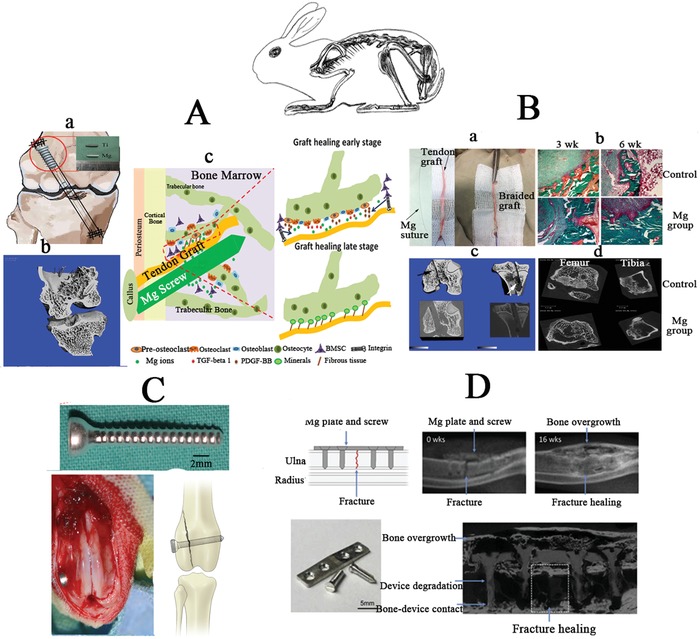
Small animal models performed in rabbits for assessing the biosafety and bio‐efficacy of Mg‐based implants. A) Mg‐based interference screw for fixation of the tendon graft in the drilled bone tunnel during anterior cruciate ligament (ACL) reconstruction in rabbits. Reproduced with permission.^46^ Copyright 2017, Elsevier. B) Mg‐based suture to braid with the tendon graft for ACL reconstruction in rabbits. C) Mg‐based screw applied for fixation of bone fractures in the distal femur of rabbits. Reproduced with permission.^51^ Copyright 2015, Elsevier. D) Mg‐based screw and plate used for fixation of ulna fracture in rabbits. Reproduced with permission.^57^ Copyright 2015, Elsevier.

**Figure 7:**
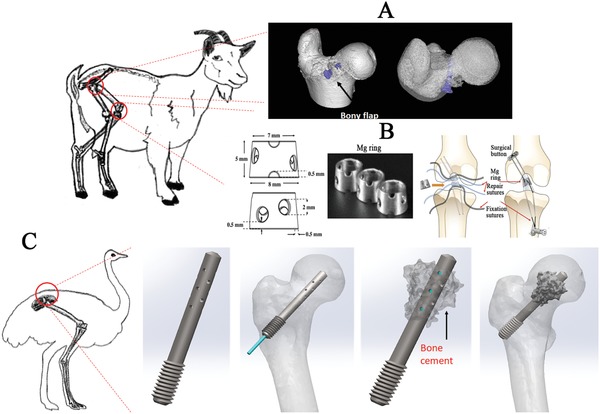
Large animal models for assessment of biosafety and bioefficacy of Mg‐based implants. A) Mg‐based screw applied for fixing the vascularised bony flap in the femoral head of goats. B) Mg‐based ring used to repair ACL of goats. Adapted with permission.^86^ Copyright 2016, Orthopaedic Research Society, Published by Wiley Periodicals, Inc. C) Mg‐based screw with holes in the shaft for injection of bone cement to repair osteonecrosis in the femoral head in emu.

As summarized in **Table** **1**, animal studies performed in both small and large animal (quadrupedal and bipedal) models, using Mg‐based implants, such as pins, plates, and screws have been extensively investigated.^17^, ^46^, ^57^, ^76^, ^77^ Most of these animal models were designed to observe the degradation pattern of Mg‐based implants and the response of peri‐implant bone tissues.^42^, ^78^, ^79^ Therefore, they do not simulate clinical scenarios, or validate potential clinical applications with regards to assessment of bioefficacy of the Mg‐based implants, leading to lack of information on design input as well as output to meet regulatory requirement.^80^ Herein, we summarize the representative animal models to target at clinical indications by using Mg‐based orthopedic implants for both biosafety and bioefficacy evaluation (**Figures** **6** and **7**).

**Table 1:** Representative animal studies on Mg and Mg‐based alloys as potential orthopedic implants

Mg‐based metals	Designed implants	Treatment	Control	Surgeries	Animal species	Degradation rate	Major findings	Effects of alloying elements on in vivo functions of metals	Clinical relevance	Ref.
Pure Mg	Interference screw	NA	Ti	ACL reconstruction	Rabbit	≈10% volume loss after 16 weeks	Improved tendon graft healing indicated by accelerated mineralization at the tendon‐bone interface	NA	Mimics clinical treatment in patients with severe ACL tear	46
Pure Mg	Interference screw	NA	Ti	ACL reconstruction	Rabbit	≈30% volume loss after 12 weeks	Increased fibrocartilage formation at the tendon‐bone interface	NA	Mimics surgical treatment in patients with ACL rupture	81
Mg alloy (Mg‐Zn‐Sr)	Interference screw	NA	PLA	ACL reconstruction	Rabbit	Complete degradation within 16 weeks	Increased bony ingrowth and decreased loss of the peri‐tunnel bone tissue	Increased maximum torque, lower corrosion resistance and more bone in the peri‐tunnel region	Mimics ACL reconstruction in patients	77
Mg alloy (MgYREZr)	Screw	NA	Ti6Al4V	Tendon‐bone insertion	Rabbit	≈25% volume loss after 4 weeks	Stable fixation of the tendon graft and no inflammatory reactions	Improved corrosion resistance compared to Mg‐6Zn	Mimics ACL reconstruction in patients	82
Pure Mg	Screw	NA	PLA	Femoral intracondylar fractures	Rabbit	≈30% volume loss after 24 weeks	Enhanced fracture healing	NA	Mimics intra‐articular fracture fixation	51
Pure Mg	Screw and plate	NA	Ti	Ulna fractures	Rabbit	0.40±0.04 mm per yr	Abundant bone formation around Mg devices and no difference in flexural weight of healed ulnae with Mg devices compared to intact ulnae	NA	Mimics bone fracture fixation in weight‐bearing sites	57
Mg alloy (Mg–Ag)	Intramedullary pin	NA	Stainless steel	Femoral fractures	Mouse	Complete degradation within 133 days	Increased callus formation around the fracture gap	Improved mechanical strength to support bone fractures in the heavy load sites	Mimics fracture fixation in weight‐bearing sites	75
Pure Mg	Screw	Hybrid system	Ti	Tibial fractures	Rabbit	NA	Increased callus formation at the fracture gap	NA	Mimics fracture fixation in heavy weight‐bearing sites	45
Pure Mg	Intramedullary pin	Hybrid system	Stainless steel	Femoral fractures	Rat	NA	Accelerated fracture healing	NA	Mimics fracture fixation in heavy weight‐bearing sites	18
Mg‐Zn alloys (ZX50 and WZ21)	Pin	NA	NA	Insertion into cortical bone	Rat	ZX50: complete degradation within 12 weeks; WZ21: ≈60% volume loss after 24 weeks	Improved osteoconductive properties for WZ21 pins	More ideal orthopedic materials for WZ21 with very moderate gas formation and excellent osteoconductive properties	Bone response assessment	42
Pure Mg and Mg alloy (AZ31)	Screw	NA	NA	Insertion into cortical bone	Rabbit	31.3% while 61.5% in the volume fraction of the screw head for pure Mg and AZ31 after 12 weeks, respectively	Bone growth around both screw types	Reduced in vivo degradation rate and significant bone overgrowth for AZ31 compared to pure Mg	Bone response assessment	83
Mg alloy (LAE442)	Intermedullary interlocked nail and screw	NA	Stainless steel	Insertion into bone marrow medullary cavity	Sheep	≈0.33% and 10% volume loss for nail and screw after 24 weeks	Good biocompatibility	Moderate gas formation and predominant direct bone‐to‐implant contact without alterations of bone	Local biocompatibility assessment	84
Mg alloy (Mg‐Y)	Scaffold	NA	NA	Insertion into femoral condyle	Rabbit	Over 93% volume loss after 12 weeks	No foreign‐body reaction and gas formation	Improved corrosion resistance to reduce gas formation	Potential use of implant for the repair of the bone defect	85
Pure Mg	Ring	NA	Suture	Repair of the transected ACL	Goat	NA	Improved postsurgical knee function as compared to regular suture repair	NA	Repair effects of Mg ring on the ruptured ACL	86
Mg alloy (AZ31B)	Screw	Silicon‐containing coating	PLA and Ti	Insertion into the femoral shaft	Rabbit	NA	Improved extraction torque in the coated AZ31B group when compared to other groups	Not mentioned	Bone response assessment	87
Mg alloy (Mg‐Zn‐Ca)	Pin and screw	NA	Ti	Insertion into the bone shaft of growing animals	Rat and sheep	Rat: 0.08 mm per yr; Sheep: 0.045 mm per yr	No adverse effects in a growing‐animal model	Comparatively low in vivo degradation rate for Mg‐0.45Zn‐0.45Ca implants without inducing serious gas evolution and foreign body response	Potential of biodegradable Mg‐based orthopedic implants in Children	88
Mg alloy (Mg‐Zn‐Ca)	Screw	NA	NA	Insertion into the femoral condyle	Rabbit	NA	Excellent biocompatibility and negligible production of hydrogen gas	Lower in vivo degradation rate for Mg‐5Ca‐1Zn implants compared to Mg‐5Ca implants, contributing to negligible gas formation around Mg‐5Ca‐1Zn screws in animals	Bone response assessment	89
Mg alloy (AZ31, AZ91, WE43 and LAE442)	Pin	NA	PLA	Insertion into femoral cavity	Guinea pig	NA	Increased new bone formation around Mg rods	Lowest in vivo degradation rate for LAE442 compared to other three alloys	Bone response assessment	23
Mg alloy (Mg‐Sr)	Screw	NA	Pure Mg	Insertion into femoral shaft	Rabbit	0.55±0.03 mm per yr	Significant increase in the peri‐implant bone volume and direct bone‐to‐implant contact	Increased new bone formation and significantly higher osteogenic differentiation‐associated genes in Mg‐Sr implanted bone	Bone response assessment	78
Mg alloy (AZ91)	Pin	PCL coating	Uncoated AZ91	Insertion into femoral shaft	Rabbit	0.33% while 0.05% volume loss for uncoated and coated Mg alloys	Increased new bone formation around the coated Mg‐based pins and no inflammation or necrosis	Not mentioned	Bone response assessment	90

Notes: full name of the abbreviated forms used above

NA: not applicable; Ti: titanium; ACL: anterior cruciate ligament; PLA: poly (lactic acid); PCL: polycaprolactone

As mentioned above, the traditional permanent metallic and resorbable polymeric interference screws have several drawbacks including high Young's modulus^91^ and unfavorable acidic degradation products,^92^ which may lead to bone tunnel enlargement and impaired tendon‐bone integration after ACL reconstruction, respectively. Mg‐based interference screws have recently been applied to fix the tendon graft in the drilled bone tunnel during ACL reconstruction in rabbits.^46^, ^48^, ^77^, ^81^, ^82^

The Mg ions released from the screws promoted osteointegration of the tendon graft into the bone tunnels, which was achieved through recruiting BMSCs (Figure 6A) and increased production of bone morphogenetic protein 2 (BMP‐2) and VEGF for both osteogenesis and angiogenesis, two essential coupling events in musculoskeletal regeneration.^46^, ^81^ These results encouraged the experimental method of braiding Mg‐based suture to the tendon graft prior to ACL reconstruction, aiming to improve the tendon graft healing quality (Figure 6B). Apart from ACL reconstruction, bone fracture repair in low weight‐bearing skeletal sites (i.e., femoral intracondylar and ulna fractures) were also established using relevant animal models and treated with Mg‐based screws and plates (Figure 6C,D).^51^, ^57^ Compared to the traditional fixators, Mg‐based implants were found to significantly promote bone fracture healing without inducing biosafety issues.

Large animal models may provide more convincing information before applying Mg‐based orthopedic implants in clinics. An osteonecrosis goat model was established to test the effects of Mg‐based screws on bony flap healing in the femoral head^22^ (Figure 7A). Additionally, a Mg‐based ring device was developed to repair transacted ACL in goats^86^ (Figure 7B). The Mg‐based ring significantly improves the knee stability and function, as evidenced by mechanical tests, compared to the traditional sutures. Although histological analysis was not performed to assess the graft structure, this work developed the framework for future in vivo studies to assess the potential use of this novel ring to repair ruptured ACLs. Among the large animal models, the bipedal emu with steroid‐induced osteonecrosis has been recognized as a unique model for replicating the collapsed femoral head in patients.^93^ This model proposes a novel tool to develop Mg‐based hollow screws with holes allowing the injection of bone cement to promote new bone formation in the necrotic regions (Figure 7C). The Mg‐based screw may provide the initial mechanical support for the collapsed femoral head, while the injectable bone cement may fill the gap through the screw holes to maintain, consolidate and strengthen the support of the implant. In accordance with previous studies, the Mg ions released from the Mg‐based screws during in vivo degradation may potentially prevent the femoral head collapse, a relevant predictive evidence for future clinical efficacy assessment.

## 4 Novel Strategies for Modifying Mg‐Based Orthopedic Implants

### 4.1 Novel Surface Modification Treatments

**Figure 8:**
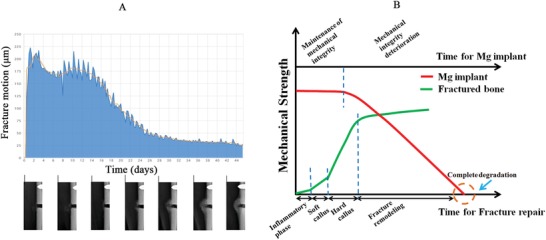
The ideal design of biodegradable Mg‐based orthopaedic implants with controlled degradation patterns to match the fracture healing. A) The description of the motion at the fracture gap with healing over time in the presence of the mechanical loading where the fracture motion at the fracture gap (upper part) decreases with healing overtime accompanied with fracture callus formation and remodeling (lower part). Adapted with permission.^98^ Copyright 2014, SCITEPRESS. B) Ideal degradation pattern of the biodegradable fixators to support healing completion at the fracture site. Reproduced with permission.^17^ Copyright 2017, Elsevier.

As the motion at the fracture gap is easily occurs under the influence of local mechanical loading at the early healing stages after surgery (**Figure** **8**A), the Mg‐based orthopedic fixators should have strong initial mechanical strength to support the fractured bone firmly at least in the early healing stages, including inflammatory phase and soft callus phase.^70^ In addition, the degradation of the Mg‐based implants may impair their mechanical integrity, so the degradation behavior of the Mg‐based implants should be optimized to match the healing process of the fractured bone (Figure 8B).

However, the mechanical strength of un‐modified Mg and its alloys is insufficient to provide initial support in high weight‐bearing skeletal sites. Although Jahn et al. reported that the use of an intramedullary pin made of Mg–silver (Mg–Ag) could successfully fix the long bone fractures without inducing pathological reactions in the internal organs, the degradation of Mg–Ag pins caused long‐term serious abnormal morphology in the long bone, which displayed over 70% increase in the bone marrow cavity diameter and relative low bone mineral density even after 133‐day implantation when compared to the stainless steel implanted bone.^75^ Surface modification technologies may open a new era in the development of Mg‐based implants suitable in high weight‐bearing skeletal sites.^94^, ^95^

Although SMAT is a surface treatment technology, a gradient distribution from several nanometers to a few micrometers is formed on the surface layer of the treated samples, contributing to dramatic improvement in the mechanical properties in both inert metals^96^ and biodegradable Mg alloys^97^ with deep treated depth. Therefore, SMAT is considered as a promising strategy to enhance the mechanical strength of Mg based alloys when used as potential orthopedic implants. However, the corrosion resistance of SMATed metals is seriously impaired due to the increase of crystalline defects such as grain boundaries and dislocations.^94^ Supra‐Nano‐Dual‐Phase alloy membrane (SNDP) via magnetron sputtering is another novel surface treatment for Mg‐based metals, which can dramatically increase their mechanical strength close to the theoretical ideal strength for the potential use in high weight‐bearing skeletal sites.^95^ However, the subsequent electrochemical reaction between the Mg substrate and the alloy coating, which acts as the second phase, can accelerate the corrosion rate of the Mg‐based metal, resulting in a rapid loss of mechanical strength.

**Figure 9:**
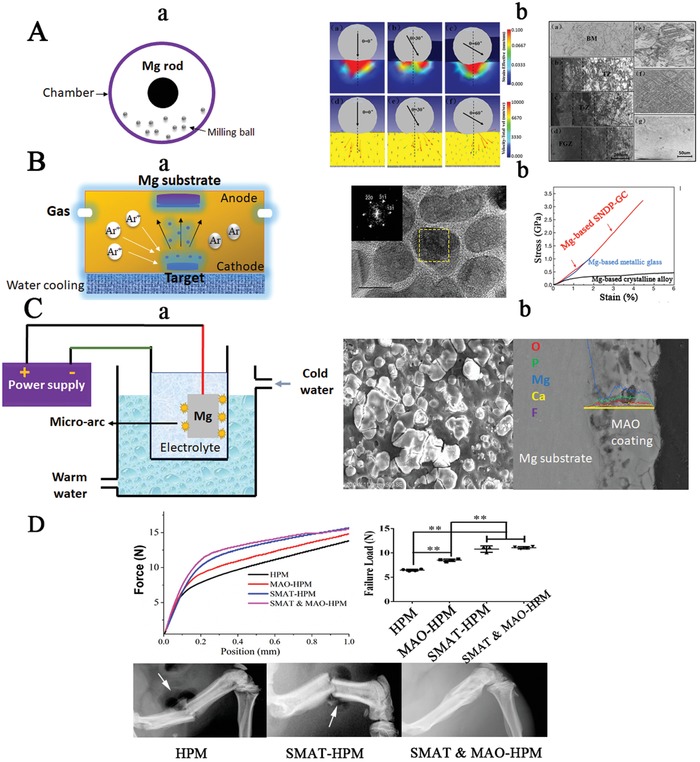
Novel strategies for developing Mg‐based implants with desirable mechanical strength and corrosion resistance. A) Surface Mechanical Attrition Treatment (SMAT) applied to enhance the mechanical strength of Mg‐based metals: (a) Schematic diagram displaying SMAT technology to coat Mg‐based metals; (b) Stain and material flow during the impact process with milling balls and the microstructure of SMATed and white (base material: BM) regions treated at different time points for observation of twinning zone (TZ), transition zone (T‐Z), and fine grain zone (FGZ). Reproduced with permission.^101^ Copyright 2019, Elsevier. B) (a) deposition of Supra‐Nano‐Dual‐Phase (SNDP) alloy membrane on the surface of Mg‐based metals by a magnetron sputtering process; (b) Observation of the dual‐phase Mg alloy nanostructure with dramatic improvement in the mechanical strength. Reproduced with permission.^95^ Copyright 2017, Nature. C) (a) Micro‐Arc Oxidation (MAO) treatment of Mg‐based metals (b) to form a Ca‐P coating (confirmed by Energy Dispersive X‐ray analysis (EDAX)) to improve corrosion resistance; D) combined surface treatment strategies may improve the mechanical strength and degradation rate at high weight‐bearing skeletal sites. Closed fractures in the femur of rats were successfully fixed by intramedullary pure Mg pins with complex coatings. The gas formation was indicated by the white arrow. HPM: high‐purity Mg.

In order to improve the corrosion resistance of Mg‐based metals treated with either SMAT or SNDP, the development of a complex surface coating strategy may be necessary to develop clinically available Mg‐based orthopedic implants that possess both excellent mechanical strength and a suitable degradation rate (**Figure** **9**A,B). Polymer coating treatment has been commonly used to reduce the corrosion rate of Mg‐based implants.^90^ However, as the connection between the polymer coating and the metal substrate was formed via the physical interaction by immersion or dipping method, the coating may be easily detached from the metal when inserted into the bone, making it less feasible for clinical application.^99^ In contrast, a chemical surface modification may be more preferable to ensure that the coating is not removed before implantation is completed owing to the strong chemical bonds at the substrate‐coating interface.^99^ Microarc oxidation (MAO) treatment is such an effective chemical modification method to improve the corrosion resistance of Mg‐based metals by forming a porous structure with strong adhesion on the substrate surface^100^ (Figure 9C). Therefore, a dual coating of SMAT or SNDP with MAO may provide a promising strategy to develop Mg‐based implants that may support high weight‐bearing skeletal sites and ensure that the corrosion rate matches the rate of bone healing (Figure 9D).

### 4.2 Other Strategies

Generally, Mg alloys corrode faster than high purity Mg because of microgalvanic acceleration of the corrosion of the alpha Mg matrix by precipitated second phases,^102^ indicating that purification in Mg matrix can slow down the corrosion rate.^103^ However, insufficient mechanical strength greatly limited the use of pure Mg metals. Novel alloying systems with appropriate amount of nontoxic elements (e.g., Ca, Sr, Zn, Si, etc.) added as binary or ternary series alloys can effectively improve the mechanical strength via refining the microstructure of Mg matrix.^24^ For example, the addition of 4% Zn into Mg can dramatically improve ultimate tensile strength from 21 MPa (as‐cast pure Mg) or 100–140 MPa (as‐extruded or as‐rolled pure Mg) to 220 MPa (as‐cast state).^24^ However, the precipitated intermetallic phase Mg‐*x*Zn may impair the corrosion resistance, so the development of the ternary alloy series may be feasible to guarantee the performance in both mechanical and degradation properties. For example, 0.2% Ca addition could reduce approximately one third of the degradation rate of as‐cast Mg‐4Zn alloy.^24^ In addition, the optimization in fabrication for ultrafine‐grained^104^ or glassy^43^ structure of Mg or its alloys can be also considered as important methods for improving mechanical strength and corrosion resistance.

## 5 The Potential Use of Mg‐Based Hybrid Implants at High Weight‐Bearing Sites

In addition to aforementioned methods to enhance corrosion resistance and mechanical strength in Mg‐based metals, the development of a Mg‐based hybrid system may be another promising strategy to develop Mg‐containing implants suitable in high weight‐bearing skeletal sites.^18^, ^45^ Our concept is to combine Mg‐based implants, which promote bone regeneration, with permanent metals that firmly fix fractures in high weight‐bearing skeletal sites.^105^ As proof‐of‐concept studies, the treatment of challenging bone diseases such as osteoporotic fractures, atypical femoral fractures, and long bone distraction has been tested in animals with innovative Mg‐based hybrid fixators.

### 5.1 Osteoporotic Bone Fracture

**Figure 10:**
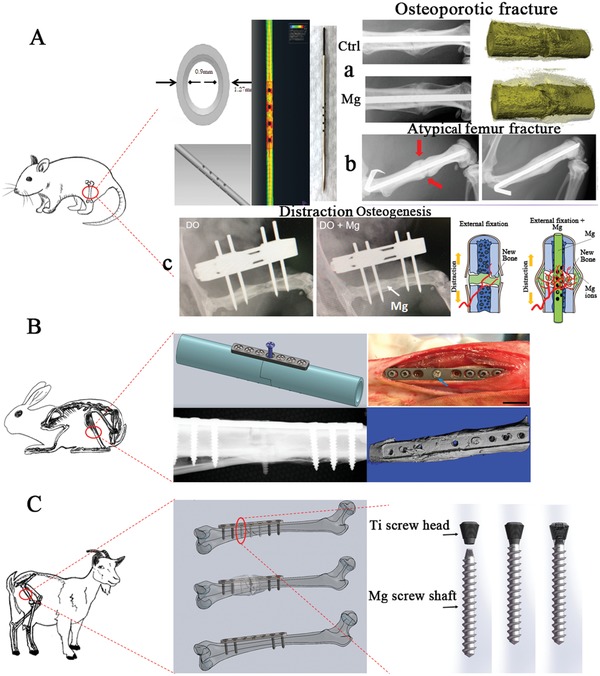
Research and development of a Mg‐containing hybrid system for application at heavy weight‐bearing skeletal regions in different animal models. A) a)Mg‐based pin‐containing stainless steel nail with holes in the steel body to allow the release of Mg ions to accelerate bone fracture healing in rats with osteoporotic fractures, b) atypical femoral fracture induced by administration of bisphosphonates, and c) distraction of open fracture. B) Mg‐based screw and titanium (Ti) plate palate‐screw hybrid system enhances bone fracture healing in tibia of rabbits. C) Mg‐based screw shaft and Ti screw head combined to promote bone fracture healing in femur of goats with osteoporotic fractures.

Osteoporotic fractures are increased with global population aging and have become a major public health burden.^106^ Recent basic research^107^ and clinical studies^108^ prove that fracture healing is impaired in osteoporotic fractures, increasing the risk of mortality due to fracture complications.^109^ It has been reported that ≈4 in 10 women over the age of 50 suffer from osteoporotic fractures in their remaining lifetime.^109^ Anti‐osteoporotic medication, including antiresorptive and anabolic agents,^110^ may be further aided by Mg‐based orthopedic implants, which provide enhanced local fracture healing as highlighted in previous sections. In order to test this hypothesis, rat models with osteoporotic fractures were used to assess the effects of a Mg‐based pin‐containing intramedullary nail on fracture healing quality (**Figure** **10**Aa). In this study, the osteoporotic fracture healing was significantly accelerated in the Mg‐treated group compared to the traditional stainless‐steel fixed group.^18^ Various Mg‐containing hybrid systems can be developed and tested in relatively larger animal models including rabbits and goats with osteoporotic bone fractures under different orthopedic conditions to mimic corresponding clinical indications, which can help justify these Mg‐containing hybrid systems for clinical trials (Figure 10B,C).

### 5.2 Atypical Femoral Fracture

Patients undergoing long‐term bisphosphonates (BPs, a commonly used anti‐osteoporotic drug) treatment are at a high risk of suffering a new kind of bone fracture, known as atypical femoral fracture (AFF).^111^ The incidence of AFF is reported to be 107/100 000 in patients who have taken BPs for more than ten years.^112^ A retrospective study placed the risk of AFF for Asian women at eightfold higher than that of Caucasian women.^113^ Recent evidence has demonstrated that individuals receiving a high dose of BP experience severely suppressed bone turnover, compared to individuals receiving a lower dose to treat bone malignancy.^114^ BP‐induced AFF typically presents with delayed fracture healing, even nonunion, when fixed with a traditional bioinert intramedullary nail (IMN) as recommended by the American Society for Bone and Mineral Research.^112^ BPs reduce the production and release of CGRP in dorsal root ganglion (DRG),^115^, ^116^ accompanied by suppressed sensory nerve sprouting in bone tissue.^115^ In addition, BPs are known for their role in inhibiting the function of macrophages,^117^ which secretes CGRP and promotes bone healing as previously discussed.^118^ Therefore, it is expected that when BPs accumulate over time, the release of CGRP from sensory nerve endings that are distributed in the periosteum, is suppressed. An AFF rat model, receiving weekly injections of BP (0.3 mg kg^−1^) for 3 months was used to test the feasibility of using Mg‐IMN for fracture fixation. The results suggest that Mg‐IMN can achieve biological healing of AFF (Figure 10Ab) through promoting osteogenesis and angiogenesis, two key factors for successfully facilitating bone regeneration and fracture healing.

### 5.3 Long Bone Distraction

Ilizarov's principle suggests that new bone tissue can be generated to fill a bone defect and help restore the bone length, with the aid of external fixators to pull the shorted bony ends apart.^119^ This technique is named distraction osteogenesis (DO). Even though DO has been applied to effectively treat bone defects for short bones such as the metacarpal and mandible,^120^ when treating lesions occurring in long bones such as the femur and tibia,^121^, ^122^ a relatively long period is required to allow new bone tissue fully mineralise and consolidate, with evidence demonstrating that patients need to bear the external fixators for more than 20 months. Concomitant complications such as nail infection, rupture, or loosening of the fixators have largely limited its application. Poor blood supply of the new tissue at the defect site after DO is one possible reason for the delayed consolidation of the new bone tissue.^122^ To avoid these complications, a pure Mg pin was implanted into the marrow cavity of the shortened bony ends during DO in a goat model (Figure 10Ac). In the femoral distraction rat model (5 mm length), improved bone tissue formation and consolidation were observed in the Mg treatment group, as compared to the traditional DO control group. These improvements may be attributed to the recapitulation of the nerve‐vessel‐bone network, regenerating bone functionality.

## 6 Current Clinical Use of Mg‐Based Implants

**Figure 11:**
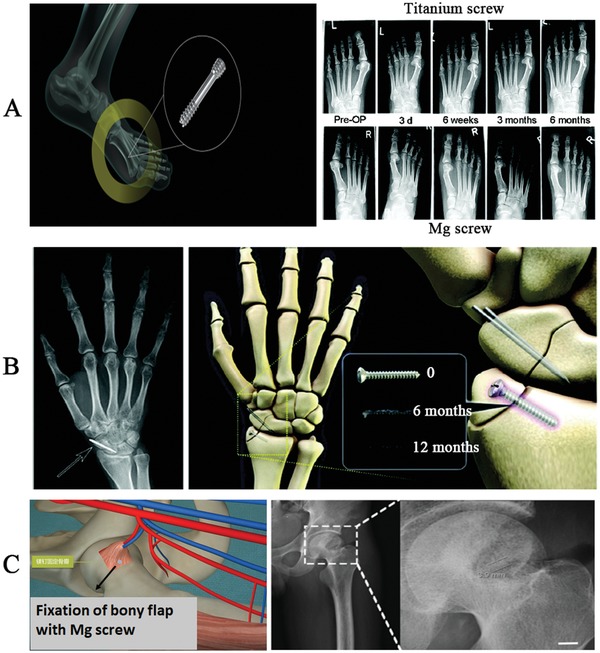
Clinical use of Mg‐based screws in orthopaedics. A) Mg‐Y‐RE‐Zr alloy screws were applied to treat a mild hallux valgus fracture in thirteen patients, with a 6‐month follow‐up for observation and assessment. Mg‐based screw had an equivalent outcome compared to the Ti control group based on the American Orthopaedic Foot and Ankle Society clinical rating score for hallux, visual analogue scale for pain assessment and range of motion of the first metatarsophalangeal joint. B) Mg‐based alloy composed of Mg‐Ca‐Zn alloy screws were used to fix the distal radius fracture in 53 patients with a one year follow‐up for observation and assessment. New bone completely replaced the biodegradable Mg‐based implant at one‐year follow‐up. Reproduced with permission.^124^ Copyright 2016, National Academy of Sciences. C) High‐purity Mg screws were developed to fix the vascularized bony flap for hip preservation treatment in 23 patients with one year follow‐up observation and assessment. The use of Mg screws significantly reduced the displacement of the bony flap and improved the healing quality according to the Harris hip score evaluation. Reproduced with permission.^125^ Copyright 2016, Elsevier.

The history of Mg‐based orthopedic implants dates back a century, but the relatively high degradation rate, impurities, and undeveloped processing techniques of Mg‐based implants inhibited their further clinical application, until recently.^17^, ^123^ Clinical trials have been conducted in Germany,^124^ South Korea,^125^ and China.^22^ Germany was the first country to use MgYReZr alloy screws (MAGNEZIX fabricated by Syntellix AG) in hallux valgus surgery (**Figure** **11**A). Between the Mg and Ti group at six month postoperation, comparable results were observed for pain assessment and range of motion of the first metatarsophalangeal joint, using the American Orthopedic Foot and Ankle Society clinical rating visual analogue scale for hallux.^124^ This clinical trial allowed the MgYReZr screw to be approved with the Communauté Européenne (CE) mark in 2013. Up to date, the clinical use of MAGNEZIX series screws has been extended to over 50 countries/regions. In China and USA, the application of the multicenter clinical trials for MAGNEZIX series screws is still under preparation. South Korea U&I company developed the K‐MET screws made of MgCaZn alloy (Figure 11B) for distal radius fracture repair.^125^ The fractures were completely healed at six months postfixation,^125^ leading to the approval of MgCaZn screws for clinical use by the Korea Food and Drug Administration in April 2015. Considering the potential health risks of the alloying elements in patients, China has focused on the development of a 99.99% high‐purity Mg orthopedic internal fixation implant (fabricated by Eontec in Dongguan, Guangdong).^126^ These pure Mg screws have been used to fix autologous vascularised bony flaps to treat avascular necrosis of the femoral head in patients (Figure 11C), demonstrating long‐term (12 months) efficacy.^22^ These screws have also been successfully used to fix the vascularized iliac graft for displaced femoral neck fracture in young adults, providing better outcomes than the control group fixed with conventional implant while with a lower rate of complications, such as avascular necrosis and nonunion.^127^ On 1 July 2019, the pure Mg screws was officially approved by China National Medical Products Administration (NMPA) for the multicenter clinical trials in the treatment of the steroid‐induced osteonecrosis (Figure 1, Supporting Information), which is the key step for the product registration of the Class III medical devices.

## 7 Challenges and Outlook

Although the biodegradable Mg‐based metals have been accepted as the potential orthopedic implants by clinicians, there are still some challenges for the commercialization of the Mg‐based implants especially for weight‐bearing skeletal sites.

First, the degradation mode of the Mg‐based orthopedic implants should be optimized. Most recently, some researchers have found that there are cavities surrounded by the fibrous tissue in the peri‐implant space after the degradation of the Mg‐based screws.^128^ As the formation of the cavities are accompanied with inflammatory responses, in which a high amount of macrophages are detected,^128^ the cavity formation may be attributed to the combined contributions of the released gas and the detached intermediate degradation products of the Mg‐based implants. As faster degradation rate of the Mg‐based implants leads to more accumulation of both gas and detached particles, the improvement of corrosion resistance of Mg‐based implants may facilitate reducing the formation of the fibrous cavities. The addition of inorganic or organic coatings on the surface of Mg‐based metals is widely considered as an effective strategy to control the degradation rate of Mg‐based implants.^24^ In agreement with this, it has been reported that negligible cavities were observed in the coated Mg‐based implants.^128^

Second, innovative design should be explored to address the concerns in the insufficient mechanical strength of the Mg‐based implants. The breakage of the screw head may sometimes happen during the operation, indicating the requirement of new structural design for the Mg‐based orthopedic implants to meet the needs of a higher torque for future clinical use.^77^

Third, more biosafety tests should be performed to identify the subjects suitable for the use of the Mg‐based implants. The stages of chronic renal failure should be classified in animal models to mimic patients with different glomerular filtration rate (GFR) levels for clear identification of indication or contraindication. In addition, the long‐term biosafety assessment of Mg implantation in animals with renal failure is lack, which should be supplemented in future studies to address clinical concerns.

Fourthly, in addition to the metallic implants, the Mg‐containing polymer‐ or ceramic‐based scaffold can be developed to promote healing in segmental bone defects through inducing and accelerating new bone formation in the presence of bioactive Mg ions^77^.

Finally, the Mg‐containing hybrid systems should be explored to extend the clinical indications. The combined use of Mg‐based implants and the inert metals can largely expand the application scope, including low and high weight‐bearing skeletal sites, which can greatly push the Mg‐involved implants forward into clinical use in orthopedic field.

## 8 Conclusions

Mg‐based implants have been successfully developed and applied in a series of clinical trials attributed to their superior biocompatibility with natural cortical bone, appropriate Young's modulus, and biodegradation into products with osteopromotive properties. Numerous animal studies have been conducted to validate the beneficial effects of Mg‐based implants on bone tissue healing, with encouraging results demonstrating that these implants may revolutionize the treatment of challenging bone diseases including osteoporotic fracture, osteonecrosis, atypical femoral fracture, and long bone distraction in patients. Surface modification strategies and Mg‐based hybrid systems may broaden the potential applications of Mg‐based implants to treat challenging bone diseases, particularly in heavy weight‐bearing skeletal sites. By understanding the physical, chemical, and biological constraints of Mg and its alloys, to identify methods for overcoming these constraints, suitable Mg‐based implants shall be developed for various clinical indications. These studies have established translational potentials by bridging the gap between basic research and clinical applications on Mg‐based orthopedic implants, with the goal to improve the well‐being of patients suffering from challenging bone diseases.

## Conflict of Interest

The authors declare no conflict of interest.

## Supporting information

Supporting Information
