# Supplementary material for: Biodegradable Magnesium‐Based Implants in Orthopedics—A General Review and Perspectives
Source: Adv Sci (Weinh). 2020 Feb 28;7(8):1902443. doi: 10.1002/advs.201902443 (PMC7175270; doi:10.1002/advs.201902443)
Supplement: Supplementary file 1 — Supporting Information [file ADVS-7-1902443-s001.pdf]

Copyright WILEY-VCH Verlag GmbH & Co. KGaA, 69469 Weinheim, Germany, 2020.

ADVANCED SCIENCE Open Access

Supporting Information

for *Adv. Sci.,* DOI: 10.1002/advs.201902443

Biodegradable Magnesium-Based Implants in Orthopedics—A General Review and Perspectives

Jia-Li Wang, Jian-Kun Xu, Chelsea Hopkins, Dick Ho-Kiu Chow, and Ling Qin**

国家药品监督管理局

医疗器械临床试验批件

批件号：2019L0005

申请人	东莞宜安科技股份有限公司
申请人住所	东莞市清溪镇银泉工业区
试验用医疗器械名称	可降解镁骨内固定螺钉
试验用医疗器械型号、规格	见附页。
试验用医疗器械结构及组成	该产品由纯度为99.99wt.%的挤压态纯镁棒材经机加工制成。经辐照灭菌，有效期为三年。
审批意见	申请人提交了试验产品描述、实验室研究资料、动物实验资料、文献资料、临床试验受益与风险分析报告、产品技术要求、注册检验报告和预评价意见、说明书及标签样稿、临床试验方案、伦理委员会同意临床试验开展的书面意见。经审查，该临床试验受益大于风险，同意其开展临床试验。
主送	/
抄送	/
备注	请于所获批临床试验方案经临床试验伦理委员会批准之后再开展临床试验。

审批部门：国家药品监督管理局　　批准日期：二〇一九年七月一日

Supplementary Fig. 1 The authorized notification from National Medical Products Administration (NMPA) in China for multi-center clinical trials with high-purity Mg screws for hip preservation in patients with osteonecrosis.
